# Endo-Wheat for ascending aortic intramural hematoma: Endo-Wheat versus endo-Bentall

**DOI:** 10.1016/j.xjse.2025.100082

**Published:** 2025-10-08

**Authors:** Mehrdad Ghoreishi, Aakash Shah, Nish Patel, Brian Schiro, Constantino Pena, Bradley S. Taylor, Tom C. Nguyen, Shahab Toursavadkohi

**Affiliations:** a Division of Cardiac Surgery, Department of Surgery, Miami Cardiac and Vascular Institute, Miami, Fla; b Division of Vascular Surgery, Department of Surgery, University of Maryland School of Medicine, Baltimore, Md

**Figure:**
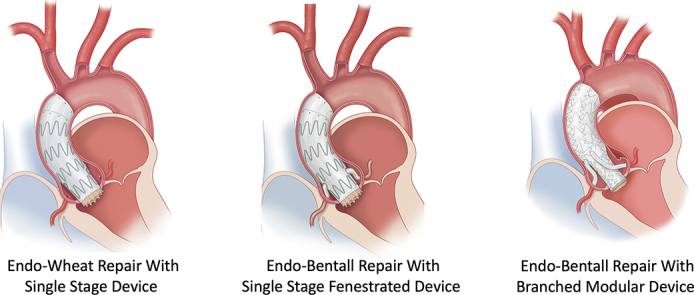
Techniques for endovascular aortic root repair.

**Central Message**

In high-risk patients with ascending aortic intramural hematoma and penetrating ulcer located in proximity of the sinotubular junction, an endo-Wheat can provide a safe landing zone.

In total, 5% to 10% of acute aortic syndromes of the ascending aorta (AAS) are considered inoperable or high risk at the time of presentation.^1^ Outcomes of medical management of AAS are poor (early mortality >57%).^2^ Ascending thoracic endovascular repair (TEVAR) in AAS (in hospital mortality of 10%-15%) is associated with favorable outcomes compared with medical management.^3^^,^^4^ In total, 40% of patients with AAS have the primary entry tear, or penetrating ulcer (PAU), within the aortic root or <1 cm distal to the sinotubular junction (STJ).^3^^,^^4^ This complicates endovascular approaches for treatment, because a proximal seal is difficult to achieve with TEVAR. The outcomes of placing an isolated ascending TEVAR in patients with a tear in proximity of aortic root are poor, with 1-year survival rate of only 30%.^3^^,^^4^ The rate of type 1a endoleak among these patients is high (40%-50%).^3^^,^^4^ Therefore, there is an unmet need for endovascular techniques that offer a safer proximal landing zone for TEVAR.

## Case Presentation

**Figure 1:**
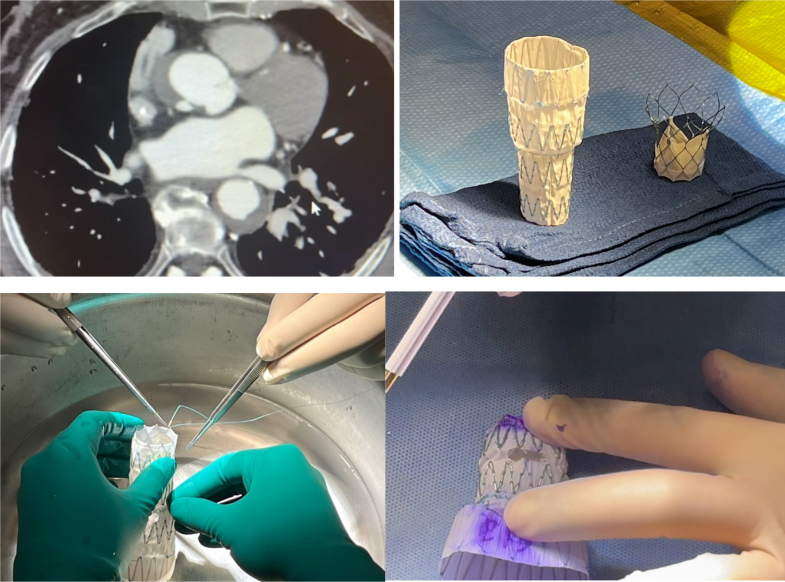
Preoperative computed tomography angiography scan. Thoracic endovascular graft and self-expanding transcatheter valve are hand-sewn together. Fenestration is created in each cusp (3 total).

A 79-year-old woman with chronic obstructive pulmonary disease and frailty, on home oxygen, with recurrent stage IV lung adenocarcinoma, and receiving chemoradiation (survival >1 year) presented with chest pain. Computed tomography angiography (CTA) scan demonstrated a 1.7-cm PAU located 7 to 10 mm above STJ (Video 1) with a 1.2-cm intramural hematoma (IMH) along the lateral curve of the ascending aorta (Figure 1, Video 1). Transthoracic echocardiography showed no aortic insufficiency and acceptable biventricular function. In a multidisciplinary clinical evaluation, open repair was deemed high risk because of the patient's frailty and advanced lung cancer. An endo-Wheat procedure was considered. Informed consent was obtained from the patient regarding publication of her study data. Institutional review board approval was not required. This study was conducted in accordance with the ethical standard of the institutional research and ethical committee.

### Physician-Modified Endo-Wheat Device Design

The design has been described previously.^5^ To summarize in brief, It is a modular system designed for “off-the-shelf,” consisting of (1) a self-expanding transcatheter aortic valve (TAVR) valve, (2) a TEVAR, and (3) wire-reinforced fenestrations. Balloon-expandable valves cannot be used with this technique because of space limitations in the delivery sheaths. Any self-expanding TAVR valve can be used. Any thoracic endovascular stent graft can be used. The TEVAR delivery sheath should be large enough to accommodate both TAVR and TEVAR (we use the Cook TX2 delivery system with size 22-Fr delivery sheath).

1. The TAVR valve is incorporated into an appropriately sized tapered TEVAR graft and the 2 components are sutured together circumferentially.
2. The left and right coronary fenestrations are made in the composite device on the basis of CTA measurements and wire-reinforced with segments of nitinol guidewire.
3. Another fenestration is made in the noncoronary cusp of the designed device. This way, all sinuses are perfused. This fenestration is to prevent any coronary malperfusion just in case the device is deployed with malalignment.
4. A radio-opaque marker is placed at the nonright commissure of the TAVR valve to facilitate positioning during deployment. Radiopaque markers that are mounted on any marker pigtails can be used.
5. The Endo-Wheat composite device is then resheathed within the TEVAR delivery system. During mounting on the TEVAR delivery system, the 3 bars that are located at the end of the TEVAR delivery system are carefully passed through the composite valve and are secured through the TEVAR delivery sheet nose-cone holes.
6. The nose-cone of the TEVAR delivery system is then trimmed from 8 cm to 4 cm so that it does not perforate the left ventricle. This is performed using surgical eye cautery.
7. The nose-cone is then reshaped with a sharp knife to become cone shaped

### Operation

A 25-mm self-expanding TAVR (Navitor TAVI System; Abbott) was chosen (aortic annuls perimeter 65.8 mm and area 337.7 mm; Video 1). The diameter of STJ and distal ascending aorta were 31 mm and 32 mm, respectively. The ascending aortic length from the aortic annulus to the innominate take-off was 9 cm in the outer curve. A tapered TX2 Cook graft, size 34 (distal) and 28 (proximal), was trimmed from an original length of 15.9 cm to 9 cm. we trimmed the graft from the proximal part, but it can be trimmed from both ends. The TAVR was hand sewn to the smaller diameter side of the TEVAR graft (28 mm side) TX2 Cook TEVAR graft using a 4-0 ETHIBOND suture (Ethicon; Figure 1, Video 2). We did not oversize the TAVR valve, because the TEVAR graft itself added more bulk to the valve. Large fenestrations (7-8 mm in diameter) were made in the left coronary cusp, right coronary cusp, and noncoronary cusp of the device. The left and right coronary height from the aortic annulus was 8.4 mm and 15.4 mm, respectively. Each fenestration was made within a 10-mm distance from the proximal edge of the endo-Wheat device (Figure 1, Video 2).

### Device Delivery and Deployment

The femoral arterial access was obtained. A temporary transvenous pacing wire was placed. A Lunderquist double-curved super-stiff wire (Cook Medical) was placed in the left ventricle. The device was advanced via right femoral access and aligned in the descending aorta to make sure the non-sinus nadir marker was located laterally. The device was than advanced and positioned 4 mm proximal to the aortic annulus. A pigtail was placed from the left common femoral artery in the noncoronary cusp. The cusp-overlap view was achieved. Angiography was performed to confirm that the nonsinus nadir marker was aligned with the pigtail and the left and right coronary fenestrations were located away from the pigtail. After rapid pacing, the device was deployed successfully (Video 2). Final angiography showed no endoleak, adequate coronary blood flow, and no aortic insufficiency (Video 2). TEE confirmed no prevalvular leak (Video 3), with a mean gradient 13, and normal biventricular function (Video 3). After the device deployment, she remained on sinus rhythm and was extubated on postoperative day (POD) 2. She developed uncontrolled atrial fibrillation that required intravenous amiodarone. She developed complete heart block on POD 4 that required a permanent pacemaker. The patient was ultimately discharged to a rehabilitation facility on POD 18. One month, 3-month, and 6-month follow-up CTA showed no evidence of endoleak (Video 3).

## Discussion

### Endo-Wheat Versus Endo-Bentall

**Figure 2:**
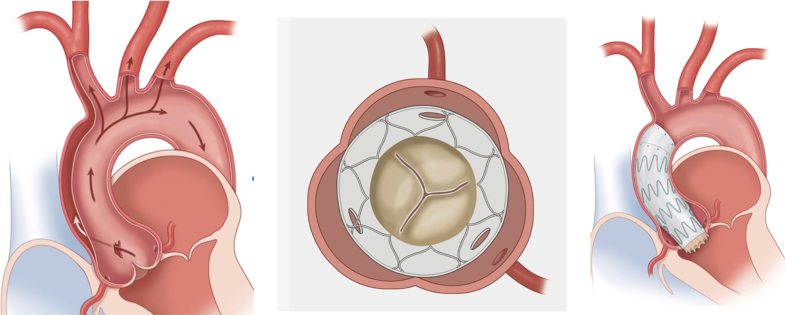
Endo-Wheat device. Thoracic endovascular aortic graft connected to the edge of the self-expanding transcatheter aortic valve and fenestration is made in each cusp.

**Figure 3:**
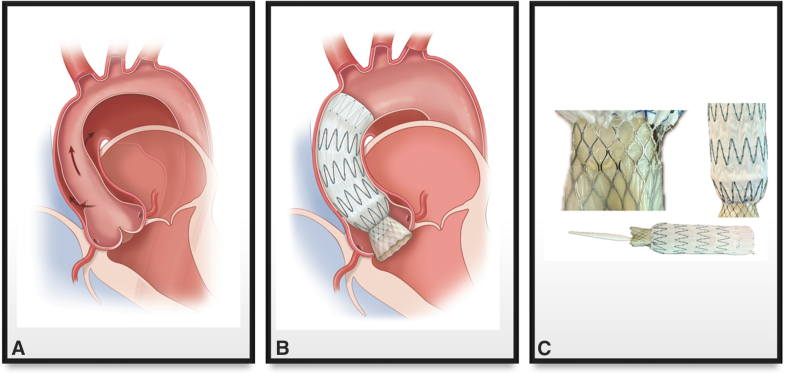
Endo-Wheat Device. A, Primary entry tear in proximity of sinotubular junction. B, Endo-Wheat repair. C, Thoracic endovascular aortic graft is sutured to the stent part of the self-expanding transcatheter aortic valve.

In endo-Wheat repair, there is blood flow to each sinus of Valsalva. The physician-modified endo-Wheat graft can be fashioned by creating fenestration in each sinus of the device (Figure 2) or by sewing the TEVAR graft to the supracommissural stent frame of the TAVR (Figure 3).

**Figure 4:**
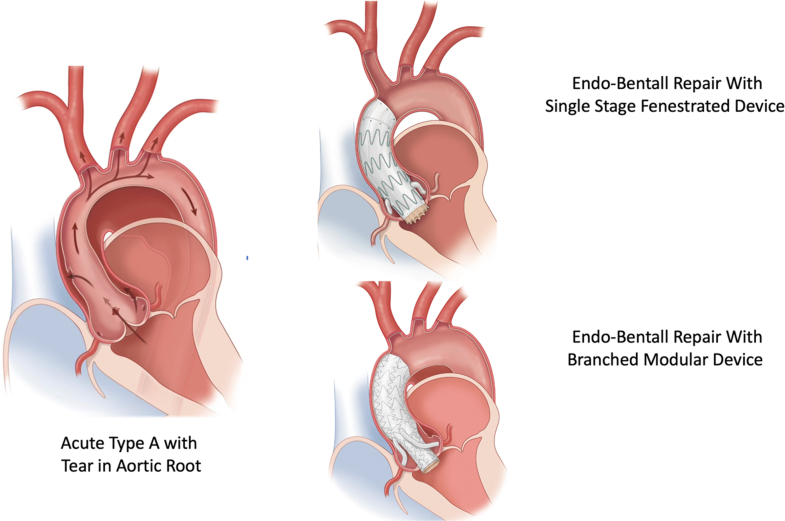
Endo-Bentall repair is shown.

In endo-Bentall repair, the fabric is present all the way to the aortic annulus. The coronary perfusion can be provided by 2 separate fenestrations (one for left main, one for the right coronary artery), or via branched grafts (Figure 4). If the PAU in IMH or aortic tear in acute type A dissection is in the proximity of the STJ (within 2 cm) and not in the aortic root, an endo-Wheat can provide a safe landing zone (Figure 2). If the aortic tear is in the aortic root, or in the case of an aortic root aneurysm, an endo-Bentall^5^ with coronary stenting is necessary to prevent an endoleak (Figure 4).

Device alignment is not critically important in endo-Wheat repair. The large fenestrations in each cusp provide blood flow to the native root. However, sealing at the STJ is important in endo-Wheat repair to prevent any endoleak from the fenestrations in the device to the entry tear. The TEVAR graft has to seal at the STJ by making sure that the TEVAR graft at the STJ is 0% to 10% oversized the diameter of the STJ. We have not seen paravalvular leak, or thrombus formation in the noncoronary cusp of the endo-Wheat device. It is important to follow these patients regularly to assure no migration of the device, no endoleak, and no prevalvular leak. Future research on endovascular aortic root repair will likely emphasize technological advancements, technique optimization, and broader clinical applications. Recapturable systems could improve device placement precision. Proximal aortic stenting may impair heart function by increasing aortic stiffness, afterload, and heart strain, potentially leading to left ventricular hypertrophy, which could restrict its use in younger patients.

## Conclusions

For patients with acute type A dissection with a tear in proximity of the STJ and not in the aortic root, or for patients with ascending aortic IMH, an endo-Wheat procedure can provide a safe landing zone.

## Conflict of Interest Statement

The authors reported no conflicts of interest.

The *Journal* policy requires editors and reviewers to disclose conflicts of interest and to decline handling or reviewing manuscripts for which they may have a conflict of interest. The editors and reviewers of this article have no conflicts of interest.

## Supplementary Data

Video 1 Preoperative CT scan. Video available at: https://www.jtcvs.org/article/S2950-6050(25)00041-5/fulltext.

Video 2 Physician-modified endo-Wheat device design. Video available at: https://www.jtcvs.org/article/S2950-6050(25)00041-5/fulltext.

Video 3 Deployment of endo-Wheat device and follow-up CT scan. Video available at: https://www.jtcvs.org/article/S2950-6050(25)00041-5/fulltext.

## References

[1] Roselli E.E., Hasan S.M., Idrees J.J. (2017). Inoperable patients with acute type A dissection: are they candidates for endovascular repair?. Interact Cardiovasc Thorac Surg.

[2] Evangelista A., Isselbacher E.M., Bossone E. (2018). Insight from the International Registry of Acute Aortic Dissection. A 20-year experience of collaborative clinical research. Circulation.

[3] Roselli E.E., Idrees J.J., Johnston D.R., Eagleton M.J., Desai M.Y., Svensson L.G. (2018). Zone zero thoracic endovascular aortic repair: a proposed modification to the classification of landing zone. J Thorac Cardiovasc Surg.

[4] Shah A., Robinson J., Chahal D. (2025). From zone 1 to zone 3: feasibility and safety of complex endovascular aortic repairs in type A aortic dissection. J Thorac Cardiovasc Surg.

[5] Ghoreishi M., Chahal D., Shah A. (2023). First-in-human endovascular root repair (Endo-Bentall) for acute type A dissection. Circ Cardiovasc Interv.

