# Supplementary figures and images for: Endo-Wheat for ascending aortic intramural hematoma: Endo-Wheat versus endo-Bentall
Source: JTCVS Struct Endovasc. 2025 Oct 8;8:100082. doi: 10.1016/j.xjse.2025.100082 (PMC13244779; doi:10.1016/j.xjse.2025.100082)

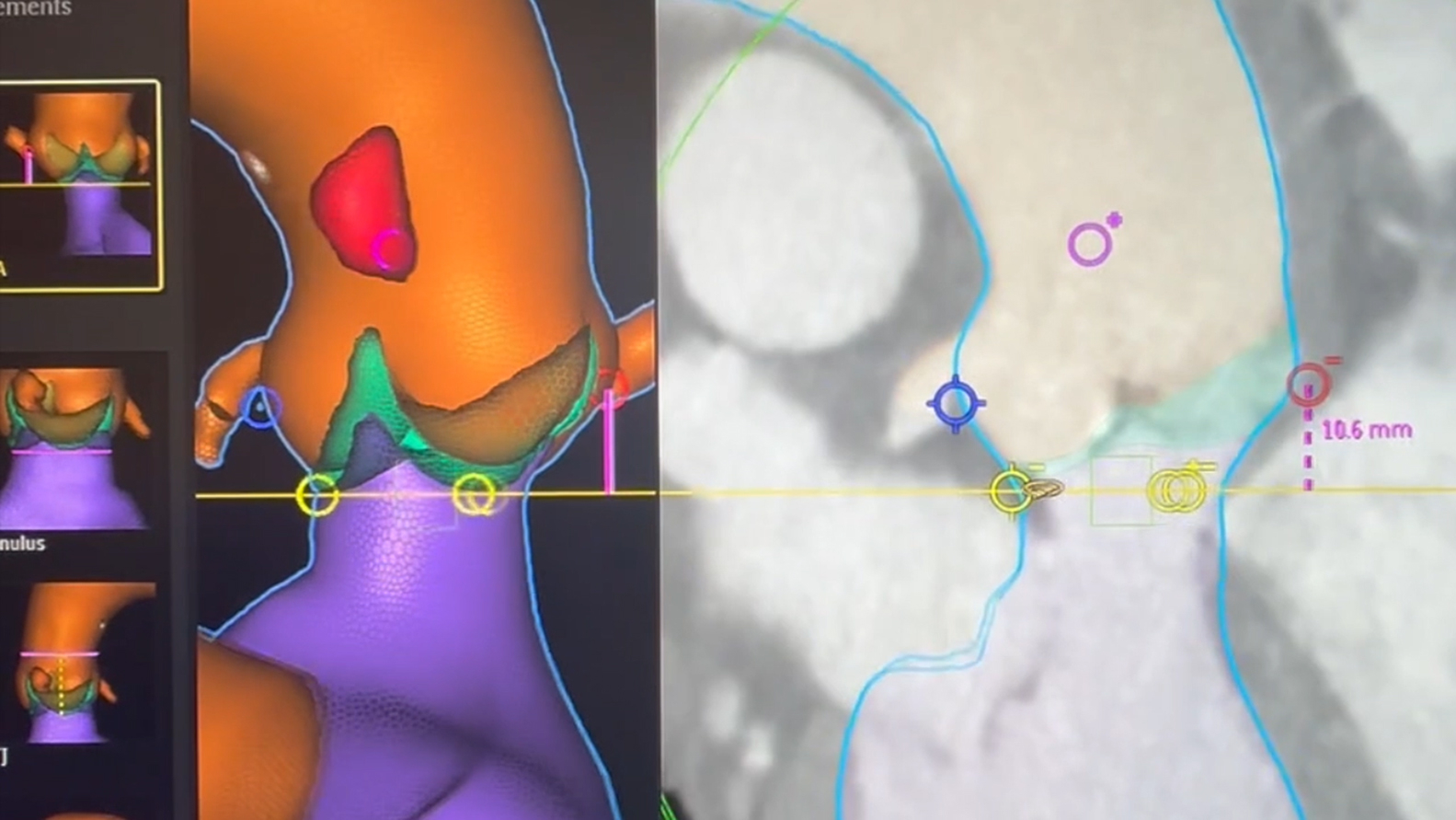

Supplement: Video 1 — Preoperative CT scan. Video available at: https://www.jtcvs.org/article/S2950-6050(25)00041-5/fulltext. [file fx2.jpg]

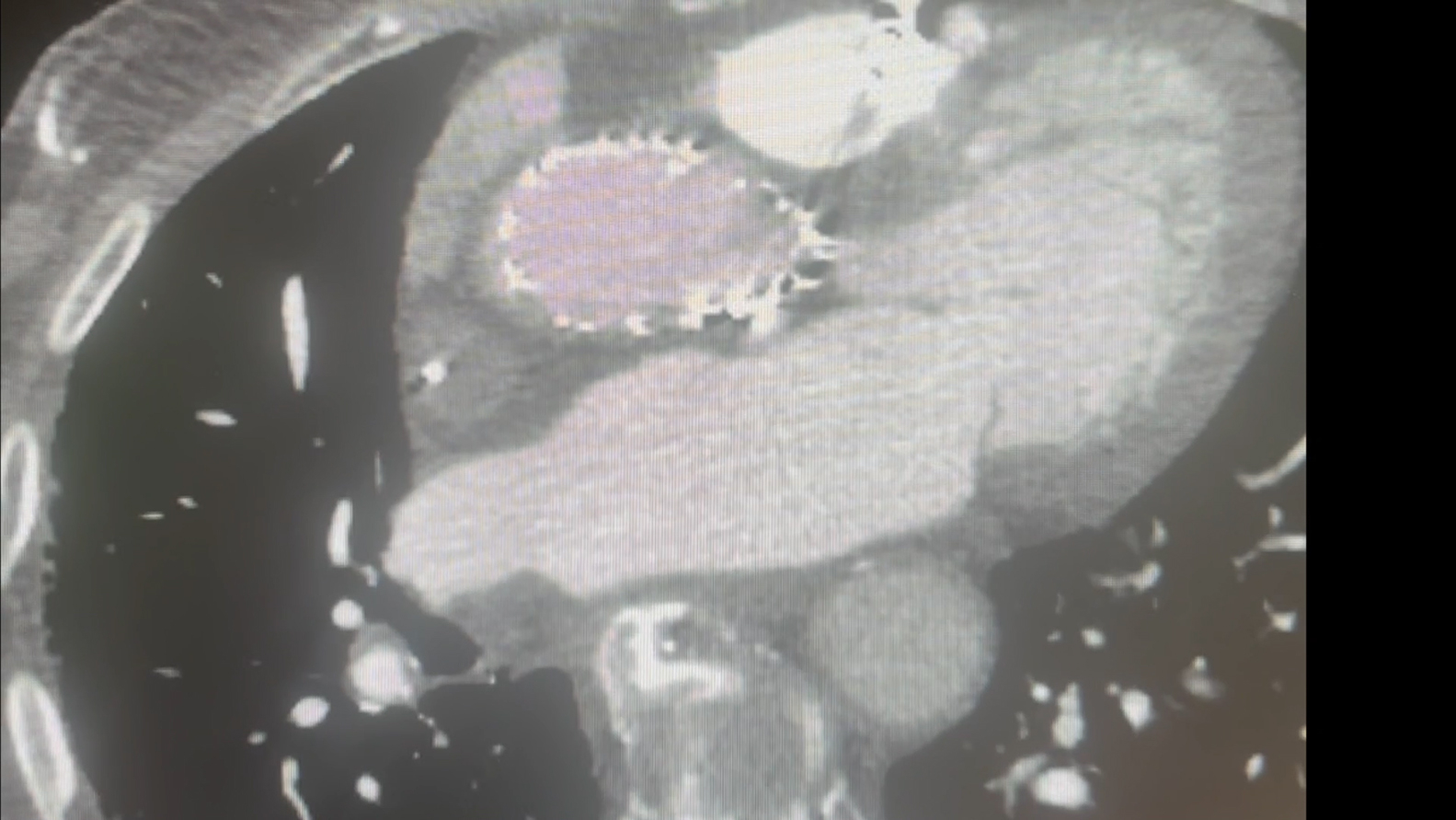

Supplement: Video 2 — Physician-modified endo-Wheat device design. Video available at: https://www.jtcvs.org/article/S2950-6050(25)00041-5/fulltext. [file fx3.jpg]

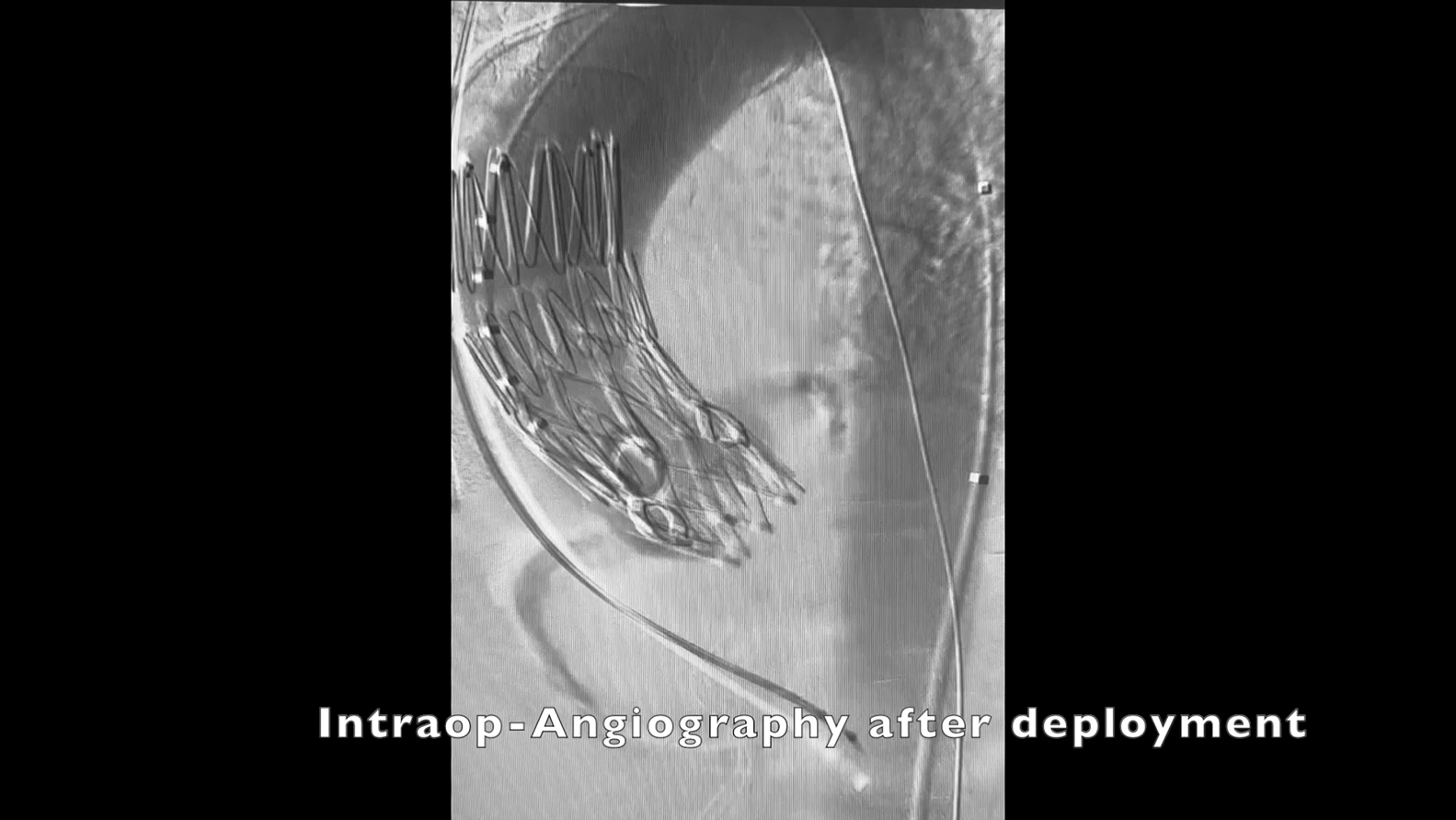

Supplement: Video 3 — Deployment of endo-Wheat device and follow-up CT scan. Video available at: https://www.jtcvs.org/article/S2950-6050(25)00041-5/fulltext. [file fx4.jpg]
